# The city of proximity (accessible, inclusive, sustainable, healthy & salutogenic): the case of Brussels Bourse, Grand-Place station

**DOI:** 10.1093/eurpub/ckac129.079

**Published:** 2022-10-25

**Authors:** G Micic, M Jevtic

**Affiliations:** 1 Faculty of Medicine, University of Novi Sad, Novi Sad, Serbia; 2 Institute of Public Health of Vojvodina, Novi Sad, Serbia; 3 School of Public Health, Université Libre de Bruxelles, Brussels, Belgium; 4 Faculty of Architecture, Architectural Engineering and Urban Planning, UCLouvain, Brussels, Belgium

## Abstract

The ecological dimension is expressed, among other things, in the matter of movement and the process of appropriation of local spaces. The creation of public space is oriented towards centralising and bringing exchanges closer together. It is a recognition of the ways of life of the individual who has become aware of the other essentials for human well-being. How does the proximity of multimodality and culture strengthen the urbanity? And how does it influence urban intensity, livability, health & the salutogenic approach of public space? The study investigates the quality of public mobility spaces through design, multimodality and sustainable planning by surveying the case of Bourse-Grand-Place station in Brussels. This transformation project is the subject of an empirical method using the material of recent research on urban design and professional practice. Falling within the scope of the “Cities for People” vision of the future, the design of this project integrates socio-cultural activities around the idea of “Station for People”. A concept based on universal accessibility ensures that all individuals can access it. Thereafter, an evolving social economy programme promoted cycling through equipment, maintenance, recycling, training, innovation and the encouragement of cycling culture. The breakthrough of the innovative multimodal design process based on multidisciplinarity could become a helpful urban strategy, oriented toward making proximate neighbourhoods both residentially and practically attractive. The present article carries out an enquiry of how design and urban activities take part in strategies to improve the quality of the public spaces. It reveals some hints that could help urban practitioners when making decisions regarding the quality of an urban place and ‘living together’ oriented developments. With a contribution to climate change issues, this article demonstrates how urban design can contribute to the quality of life of users and citizens.

